# Mitochondrial Complex IV Deficiency Nuclear Type 11 Caused by a Novel Start-Lost Variant in the *COX20* Gene

**DOI:** 10.3390/genes16091069

**Published:** 2025-09-11

**Authors:** Anna Kuchina, Artem Borovikov, Olga Sidorova, Maria Orlova, Oxana Ryzhkova, Igor Zaigrin, Aysylu Murtazina

**Affiliations:** 1 Research Centre for Medical Genetics, 115478 Moscow, Russia; borovikov33@gmail.com (A.B.); m.d.orlova@ya.ru (M.O.); ryzhkova@dnalab.ru (O.R.); murtazina@med-gen.ru (A.M.); 2 Moscow Regional Research and Clinical Institute, 129110 Moscow, Russia; sidorovaop2019@mail.ru; 3 “Biotech Campus” LLC., 117437 Moscow, Russia; izaigrin@biotc.ru

**Keywords:** mitochondrial complex IV deficiency, neuropathy, ataxia, *COX20*

## Abstract

**Background:** The *COX20* gene encodes a critical assembly factor for cytochrome C oxidase (complex IV), and biallelic loss-of-function variants in this gene cause mitochondrial complex IV deficiency, typically presenting in infancy or childhood with hypotonia, ataxia, neuropathy, or dystonia. **Methods:** This study describes an adult male patient with a broad clinical spectrum of central and peripheral nervous system involvement. Different medical genetic tests were performed for the patient, and only whole-genome trio sequencing identified pathogenic variants in the *COX20* gene. A review of previously reported cases was conducted to compare clinical and imaging findings. **Results:** Two compound heterozygous *COX20* variants in were identified: a known missense variant (c.41A>G; p.Lys14Arg) disrupting splicing, and a novel start-loss variant (c.2T>C; p.Met1?). The patient exhibited progressive ataxia, pyramidal signs, and peripheral neuropathy, accompanied by cervical spinal cord atrophy on spinal cord MRI and lower leg muscle fat infiltration on muscle MRI, an imaging feature not previously emphasized in *COX20*-related disease. **Conclusions:** A review of previously reported cases underscores broad clinical variability of the *COX20*-associated disorder, which may contribute to a prolonged diagnostic odyssey.

## 1. Introduction

Mitochondrial complex IV deficiency nuclear type 11 is a rare autosomal recessive mitochondrial disorder caused by biallelic loss-of-function (Lof) variants in the *COX20* gene. The *COX20* gene encodes a chaperone protein that specifically binds and stabilizes COX2, a catalytic core subunit of mitochondrial cytochrome c oxidase [1].

To date, approximately 31 patients with heterozygous or homozygous variants in the *COX20* gene have been reported, with most cases occurring in individuals of Chinese descent [2,3,4,5,6,7,8,9,10,11]. This predominance is linked to the high frequency of the pathogenic variant c.41A>G (p.Lys14Arg), which accounts for the majority of cases in China. Functional studies confirmed that this variant disrupts normal splicing, inducing a 20-nucleotide deletion in exon 1 and a subsequent frameshift, ultimately leading to the complete loss of COX20 protein [3]. Overall, around 10 disease-causing variants have been reported to date, predominantly missense or splice-altering variants.

In 2022, Ban et al. proposed a phenotypic classification of patients into distinct clinical subgroups based on their predominant symptomatology [4]. Early childhood-onset axonal neuropathy represents the most prevalent phenotypic subgroup. Less represented are the groups of patients with infantile hypotonia without neuropathy and neuropathy/dystonia ataxia overlapping syndrome [4]. However, subsequent clinical observations have revealed a broader phenotypic spectrum of the disease. Specifically, three patients from two Chinese families were described with previously unreported manifestations, including visual impairment, strabismus, and ptosis [5,6]. An encephalomyopathy phenotype has also been described in an adult patient with disease onset in the first year of life, presenting with developmental delay, intellectual disability, and muscle weakness. Additional features included hearing impairment, eye symptoms, movement disorder (dystonia, choreoathetosis, ataxia), and peripheral neuropathy [8].

Hereditary ataxias represent a heterogeneous group of disorders primarily characterized by progressive ataxia arising from degenerative changes in the cerebellum. Many forms of spinocerebellar ataxia, which are among the most common hereditary ataxias, are caused by trinucleotide repeat expansions in several genes (*FXN, ATXN1, ATXN2, ATXN3*) [12].

In this case report, we described the clinical manifestations of an adult patient with disease onset in early childhood, initially presenting with ataxia. Genetic analysis revealed the frequent pathogenic variant, c.41A>G (p.Lys14Arg), in compound heterozygous state with a novel variant, c.2T>C (p.Met1?), which results in the loss of the initiation codon.

## 2. Materials and Methods

Written informed consent was obtained for clinical and genetic examination and anonymous publication from the proband and his parents. The diagnostic evaluation included laboratory investigations (serum creatine kinase levels), neurophysiological studies (nerve conduction studies and needle electromyography, Neuro-MEP-micro, Neurosoft, Ivanovo, Russia), and magnetic resonance imaging (brain, spinal cord, lower limb muscle MRI, GE Medical Systems, Optima MR450w, Waukesha, WI, USA).

DNA was extracted from whole blood samples using a Wizard^®^ Genomic DNA Purification Kit (Promega, Madison, WI, USA). A custom AmpliSeq™ panel comprising 21 genes was used for sequencing on an Ion Torrent S5 next generation sequencer as described earlier [13]. The whole genome sequencing data was obtained as part of a collaboration with “Biotech Campus” (National Genetic Initiative “100,000 + Me”). The Initiative aims to genotype 100,000 individuals from varied Russian geographic and ethnic populations. WGS was performed using a DNBSEQ-T7 instrument in a pair-ended mode (2 × 150 b.p.) with an average on-target coverage of 30× with MGIEasy FS PCR-Free DNA Library Prep Set (MGI Tech Co., Shenzhen, China) for library preparation (Biotech Campus, Moscow, Russia). Sequencing data was processed using the online service for automated bioinformatics analysis of high-throughput sequencing data “NGSData”. The transcript NM_198076.6 was used as a reference for variant annotation in the *COX20* gene.

## 3. Case Report

**Figure 1:**
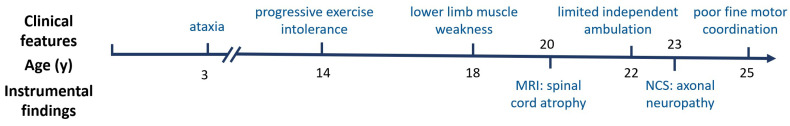
A timeline illustrating the progression of the patient’s symptoms.

The proband is a 26-year-old male whose chief complaints included difficulty maintaining balance while walking and standing, slow speech, and bilateral foot deformities. He was born to non-consanguineous Russian parents, with one healthy older brother in the family. The patient was born at 39 weeks gestation from a fifth pregnancy complicated by maternal anemia, exacerbation of chronic pyelonephritis, and chronic fetal hypoxia. The neonate’s birth parameters included a weight of 3750 g, length of 53 cm, and Apgar scores of 7/8. Motor milestones were achieved within normal ranges, including head control at 2 months, sitting at 7 months, and unassisted walking at 13 months. Mental and speech development were consistent with age-appropriate milestones. Parents first noticed gait instability at the age of 3 years (Figure 1). From an early age, the patient demonstrated persistent hyperexcitability and aggressive tendencies. He also reported progressive exercise intolerance since age 14, with complete cessation of running by age 18 due to lower limb muscle weakness. Since the age of 22, the patient has required a cane for ambulation. In recent years, he has developed progressive bilateral hand clumsiness, affecting fine motor coordination.

**Figure 2:**
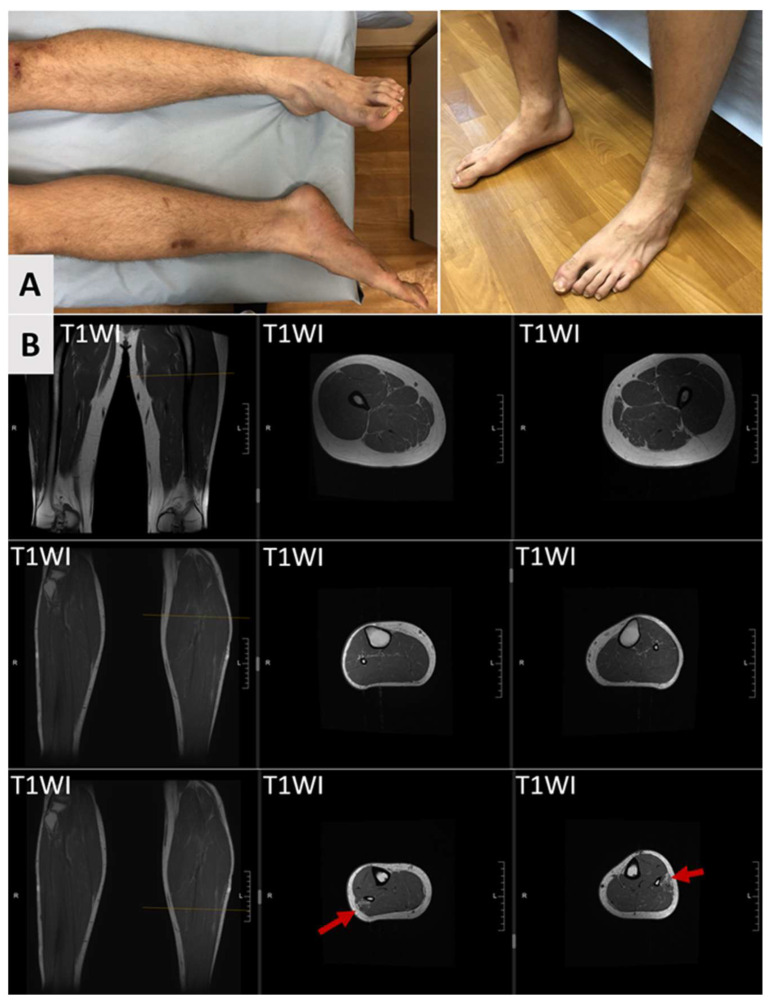
Clinical and lower limb muscle MR images of the proband. (**A**) Clinical presentation at age 26 years showing bilateral lower leg and foot muscle hypotrophy with pes planus. Also, arachnodactyly and sandal gap are noticed on both sides. (**B**) Coronal and axial T1-weighted MR images of lower limb muscles (age 20 years) demonstrate bilateral fat infiltration isolated to the peroneus longus muscles (arrows), with preservation of all other lower limb muscles.

At the age of 26, the patient exhibited phenotypic features including auricular dysplasia, arachnodactyly, dystrophic nails, sandal gap, and striae distensae on the lower back. Neurological examination revealed mild dysarthria without other cranial nerve involvement. The patient displayed extrapyramidal muscular rigidity in the upper limbs and hypotonia in the lower limbs with hypermobility of the knee joints. Mild distal leg muscle hypotrophy and pes planus were observed (Figure 2A). Deep tendon reflexes were diminished in the lower extremities, and pathologic reflexes were present in the lower limbs, though muscle strength was normal in all groups. Sensory testing demonstrated hyperesthesia, impaired proprioception, and reduced vibratory sensation in the lower extremities. Cerebellar testing revealed bilateral intention tremor, dysdiadochokinesia, a positive Romberg sign, and a severe ataxic-spastic gait disturbance requiring walking assistance. No fasciculations were observed. Thus, the patient demonstrated signs of complex involvement of both the central and peripheral nervous systems.

Electroencephalographic studies conducted showed no evidence of epileptiform activity. Electrocardiographic, echocardiographic, and ophthalmologic evaluations revealed no pathological findings. Serum creatine kinase levels were within normal limits (165 U/L, normal range: 40–200 U/L).

Nerve conduction studies demonstrated reduced amplitudes of compound muscle action potentials and sensory nerve action potentials in lower extremity nerves, consistent with axonal neuropathy. Needle electromyography revealed chronic neurogenic changes in leg muscles, in the absence of spontaneous activity.

**Figure 3:**
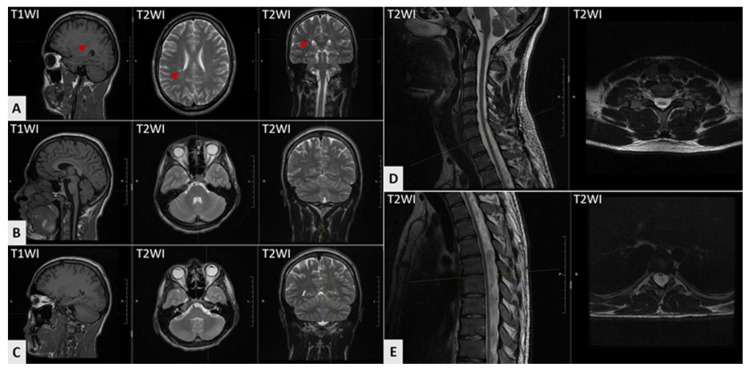
Brain and spinal cord MRI findings of the proband at ages 18 and 20 years, respectively. (**A**) A focus of increased signal intensity is visible on T2-weighted images in the right parietal lobe (arrows). (**B**,**C**) No evidence of cerebral or cerebellar atrophy is present. The brainstem appears normal without pathological changes. (**D**,**E**) Spinal cord MRI demonstrates generalized atrophy in both the cervical (**D**) and thoracic (**E**) regions.

Serial brain MRI scans obtained at the ages of 11, 15, and 18 years demonstrated a hyperintense white matter lesion in the right parietal lobe without progression (Figure 3A). Cerebellar atrophy was absent on all imaging studies (Figure 3B,C). Spinal cord MRI performed at the age of 20 years demonstrated cervical and thoracic cord atrophy (Figure 3D,E). Axial MRI scans of the lower limb muscles demonstrated mild fat infiltration in both peroneus longus muscles on T1-weighted images (Figure 2B). Thus, the instrumental diagnostics confirmed both central and peripheral nervous system involvement.

The combination of ataxic syndrome, polyneuropathy, and pyramidal signs prompted suspicion of an inherited pathology in the proband. The proband previously underwent metabolic profiling (blood amino acids/acylcarnitines, urinary organic acids), DNA testing for repeat expansion ataxias (*FXN, ATXN1, ATXN2, ATXN3*), and gene panel sequencing to search for variants in genes associated with hereditary motor–sensory neuropathies and diseases with similar phenotypes. The absence of significant findings in these diagnostic tests led to the implementation of whole genome sequencing in trio (proband and both parents).

The WGS-trio study identified two variants in the *COX20* gene (NM_198076.6) in a compound heterozygous state: c.41A>G (p.Lys14Arg) and c.2T>C (p.Met1?). The variant c.41A>G (p.Lys14Arg) has been previously reported as pathogenic in multiple studies [2,3,4,5,6,11], while the variant c.2T>C (p.Met1?) was absent from the literature and found in the gnomAD database (v4.1.1) at a frequency of 0.002088%. The variant c.2T>C (p.Met1?) was classified as pathogenic according to ACMG guidelines (PVS1, PM2, PM3). Therefore, a molecular diagnosis of mitochondrial complex IV deficiency nuclear type 11 was confirmed for the patient.

The patient was prescribed levocarnitine (1 g/10 mL), 1.5 measuring spoons (approximately 15 mL) 2–3 times daily, diluted in water and taken 30 min before meals. In addition to levocarnitine, the patient was prescribed ubidecarenone at a dosage of 30 drops twice daily for a two-month course, as well as vitamin therapy consisting of pyridoxine hydrochloride (vitamin B6, 200 mg), thiamine hydrochloride (vitamin B1, 100 mg), and cyanocobalamin (vitamin B12, 0.2 mg), administered as one tablet once daily for one month, with this course repeated twice per year. The disease progressed slowly, and the patient’s condition remained stable throughout the 2-year observation period.

## 4. Discussion

In this report, we presented a clinical case of an adult patient with a *COX20*-associated disease. To date, only 10 causative variants in the *COX20* gene total in 31 patients have been described in the literature [2,3,4,5,6,7,8,9,10,11]. In our patient, the search for repeat expansions in common spinocerebellar ataxia genes, as well as a gene panel for hereditary neuropathies, which did not include the *COX20* gene, revealed no significant findings. WGS-trio identified compound heterozygous variants in the *COX20* gene, the frequent variant c.41A>G (p.Lys14Arg) and the novel variant c.2T>C (p.Met1?).

The frequent missense variant c.41A>G (p.Lys14Arg) was reported in 26 patients, present either in a homozygous state or in a compound heterozygous state with other variants [2,3,4,5,6,10,11]. This variant disrupts normal splicing by abolishing the donor splice site of exon 1 that leads to a 20-nucleotide deletion [3]. This results in the introduction of a premature stop codon Gly8ValfsTer2 and triggers nonsense-mediated decay, ultimately resulting in the complete absence of functional protein [3,4,9]. This pathogenic variant was detected in all reported Chinese and American cases, with a shared 106 kb haplotype identified in all eight Chinese families, supporting a founder effect [3]. According to gnomAD (v4.1.1), the variant also occurs in European populations at a frequency of 0.00008559—about 5.6 times lower than in East Asian populations—suggesting it may still contribute to disease in Europeans.

The second variant identified in our proband; c.2T>C (p.Met1?) is presented in the gnomAD database (v4.1.1) with an allele frequency of 0.002088%. This variant was submitted to ClinVar twice by different clinical laboratories as a variant of uncertain significance, though no clinical information was provided (VCV001320208). Additionally, six other start-loss SNVs are present in gnomAD (v4.1.1), none of which have been reported in ClinVar or in the literature. The variant c.2T>C results in the loss of the canonical translation start codon of *COX20*. In silico analysis revealed the absence of downstream in-frame methionine codons (the full-length wild-type COX20 protein contains 118 amino acids), suggesting that c.2T>C most likely leads to a complete loss of protein expression from this allele.

Clear phenotype–genotype correlations remain difficult to establish due to limited data [2,3,4,5,6,7,8,9,10,11]. A review of the literature indicates that the primary clinical manifestations of *COX20*-associated disease include ataxia, gait disturbances, and areflexia (or hyporeflexia) (Table 1). Additionally, common manifestations of the disease, occurring in more than half of patients, also include dysarthria and motor–sensory neuropathy, characterized by muscle weakness, diminished vibration sensitivity, and abnormal nerve conduction studies. Foot deformities are also frequently observed, affecting approximately 40% of cases. Muscle tone in the extremities may vary, with some patients exhibiting hypotonia in some limbs and hypertonia in others. Ophthalmological problems and hearing loss are rarely reported. Earlier conducted studies indicate that different variants, when assessed by Western blotting, can lead to varying degrees of protein level reduction, which may contribute differently to the clinical diversity observed among patients. We hypothesize that this variability in protein expression may contribute to the differences in clinical presentation, although further research is needed to confirm direct correlations [3,4,7].

Most patients with homozygous or compound heterozygous variants c.41A>G typically present with early-onset neuropathy with a median onset at the age of three years [4]. However, our patient exhibited a distinct clinical course: disease onset characterized by slowly progressive cerebellar symptoms, with neuropathy only detected by nerve conduction studies and foot muscle hypotrophy in his adulthood.

Ataxia, as the leading clinical manifestation in our patient, warranted consideration of hereditary disorders, particularly spinocerebellar ataxias. This diagnostic hypothesis was supported by accompanying neurological features characteristic of spinocerebellar ataxias, including neuropathy and pyramidal signs, which were observed in our case. An additional key finding was cervical spinal cord atrophy. This may serve as an early and even predominant neuroimaging marker in certain spinocerebellar ataxias, while typical cerebellar atrophy might be minimally expressed or manifest only at later disease stages [14,15,16,17].

As reported in previous cases, the patient was treated with a “mitochondrial cocktail” including levocarnitine, vitamin B1, vitamin B6, vitamin B12, and ubidecarenone [4]. Although no clinical improvement was observed, the patient’s condition remained stable throughout the two-year observation period. This stability, however, could be consistent with the natural history of the disease.

## 5. Conclusions

We described a case with a novel variant in the *COX20* gene, leading to the loss of the start codon. Additionally, we conducted a review of previously reported cases, which demonstrated that the disease exhibits wide clinical variability. Nevertheless, *COX20*-related disease represents a clinical continuum, frequently including combination of ataxia, dysarthria, spinal cord atrophy, and peripheral motor–sensory neuropathy.

## Figures and Tables

**Table 1:** Frequency and distribution of clinical manifestations in *COX20*-associated disease.

Clinical Picture	Frequency
Age at examination, years (range, mean)	1–32 (16)
Age of onset, years (range, mean)	0–17 (4)
Global developmental delay	38% (14/32)
Intellectual disability	28% (9/32)
Gait disturbance	65% (20/31)
Wheelchair dependency	26% (8/31)
Could not walk without support	9% (3/31)
Ataxia	97% (28/29)
Dysarthria	58% (18/31)
Dystonia	28% (9/32)
Choreoathetosis	9% (3/32)
Muscle hypotonia	39% (12/31)
Seizures	3% (1/32)
Pyramidal signs	16% (5/31)
Limb muscle hypertonia	13% (6/31)
Peripheral motor–sensory neuropathy	53% (16/30)
Muscle weakness	53% (17/32)
Distal muscular hypotrophy/atrophy	16% (5/31)
Foot drop	23% (7/31)
Foot deformity	42% (13/31)
Sensory neuropathy	33% (10/30)
Reduced vibration sense	81% (9/11)
Hyperalgesia	27% (9/11)
Areflexia/hyporeflexia	74% (23/31)
Hyperreflexia	3% (1/31)
Hearing impairment	6% (2/31)
Ptosis	3% (1/31)
Strabismus or ophtalmoparesis	10% (3/31)
Visual impairment	13% (4/31)
**Brain and spinal cord MRI**	
MRI: cerebral atrophy	28% (5/18)
MRI: cerebellar atrophy	17% (3/18)
MRI: T2-hyperintensities at cerebral white matter	5% (1/18)
MRI: T2-hyperintensities at thalamus	5% (1/18)
MRI: spinal cord atrophy	44% (8/18)
**Nerve conduction study**	
NCS: CMAP reduced/absent	78% (18/23)
NCS: SNAP reduced/absent	100% (24/24)

MRI: magnetic resonance imaging; NCS: nerve conduction study; CMAP: compound muscle action potential; SNAP: sensory nerve action potential.

## Data Availability

The original contributions presented in this study are included in the article. Further inquiries can be directed to the corresponding author.
